# Measurement of Sorption Isotherms to Guide Mixed Display of Archaeological Iron, Bone, and Glass

**DOI:** 10.3390/ma17235934

**Published:** 2024-12-04

**Authors:** David Thickett, Antanas Mėlinis, Bhavesh Shah

**Affiliations:** English Heritage, Ranger’s House, Chesterfield Walk, London SE10 8QX, UK; bhavesh.shah@english-heritage.org.uk

**Keywords:** conservation science, archaeological iron, archaeological bone, archaeological glass, DVS, acoustic emission, FTIR, relative humidity, oxygen depletion, akageneite

## Abstract

This study examines the preservation challenges of archaeological iron, bone, and glass within shared environments focusing on material-specific degradation mechanisms. The relative humidity (RH) requirements for these materials can vary significantly. Iron presents distinct stability groups at specific RH thresholds, albeit levels below 30% RH are recommended for sensitive artefacts. Little is known about the moisture response of bone, and the heterogeneity of the material poses additional challenges for its examination. Glass can undergo deterioration at both high and low RHs due to the threat of aqueous attack and transformation of the pristine glass or the crizzling and delamination of the already transformed glass. Experiments employing dynamic vapour sorption (DVS), acoustic emission (AE), and oxygen depletion analyses provided insights into the moisture response behaviours of these materials. It was found that the deleterious akageneite formation increases dramatically at RHs > 30% in archaeological iron, reinforcing the current guidelines. Bone exhibits significant hygroscopicity as well as isotherm steepening below 25% RH on desorption, suggesting this threshold could be advisable to avoid structural damage. In glass, there is evidence of significant mass fluctuations between c. 60 and 95% RH, as well as isotherm steepening around 30% RH on desorption, thus providing more empirical evidence to published storage recommendations. This work sheds more light on the risk assessment for mixed-material showcases and underscores the necessity of nuanced RH guidelines that consider the material-specific degradation mechanisms.

## 1. Introduction

There are issues with different RH requirements of materials in the same showcase. Interpretation often requires the display of objects together of different materials to illustrate particular themes if they have particular themes or if they were excavated together. Organic and inorganic artefacts often have different requirements for preservation. The issue is most problematic when the archaeological metals iron and copper are present. Many of these artefacts require low RH conditions, and these low RHs can damage other material types. The response of museum collections to the environment is an enormous area of research. Partial summaries are given in two standards [1,2]. Whilst the RH response of several historical material is well known, that of archaeological materials is much less well researched and is investigated in this work.

The response of terrestrial archaeological iron and copper alloys has been thoroughly investigated over the past two decades [3,4,5,6,7,8,9,10,11,12,13,14,15] and is well understood, although there is some disagreement on details. The corrosion products present during and post burial have been thoroughly identified, and these analyses are summarised in Table 2.1 of [14]. Chloride introduced in burial is understood to generate the mineral Akaganeite, which has been ascribed as the cause of the significant instability observed [5,16,17,18].

For archaeological iron four behaviours have been identified.

There is material that is stable up to a very high RH; this material needs no RH control and poses no risk to material displayed alongside it. Within English Heritage’s collection, this forms about 60% of the collection.

**Figure 1:**
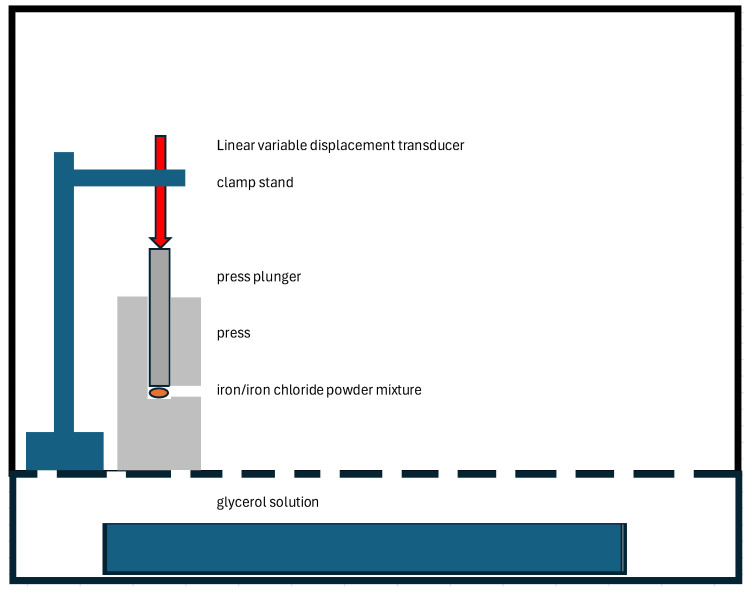
Setup to measure expansion of iron/iron chloride powders at 30 and 40% RH.

The majority of unstable material (about 85%) follows the response curve A shown in Figure 1. If the objects were stored at high RH after excavation, they can begin to react very slowly at 11% RH. Most objects will react slowly from 16% RH. Between 30 and 35% RH, the reaction rate increases by a factor of 5 [1]. There is a second large increase between 50 and 60%, the actual value depending on temperature. A series of seminars with nine archaeological curators determined that the losses likely at 30% RH were unlikely to lead to significant loss of archaeological information, whilst those at 35% likely to lead to significant loss. The 30% RH value has been adopted as a target for controlling showcases with archaeological iron internally. Some additional work was undertaken measuring oxygen depletion and expansion over this RH range to better quantify the risk. The lower value of 16% RH is used for storage.

A second group of unstable material reacts from 60 to 65% but is stable below this value. This probably has no impact on the other material displayed in the same showcase.

The final group of unstable material reacts much more quickly at 30% than group A. This material needs to be kept at RHs below 20% RH, which is problematic for many other materials.

Archaeological copper alloys have been determined to show only two behaviours pertaining to stability up to a high RH. The proportion of unstable material is much lower than archaeological iron. A reaction was found to begin at 28%, but the rate is extremely slow and unlikely to be discernible on actual artefacts within 50 years. The rate increases dramatically from 35% RH, and this has been adopted as an internal target.

Individual artefacts can be assigned to a particular behaviour group using oxygen depletion measurements [13,14,19,20,21]. In many situations, analysis of a number of potential objects can identify stable iron or copper alloy objects that can fulfil the interpretation requirements without the need for RH control. However, this is not possible in other situations, and many archaeological objects are formed of both unstable metals and other materials, requiring different RH conditions. 

The RH performance of a showcase depends on the room environment, its air exchange rate and the loading of buffer materials. Both intentional buffers such as silica gel and many dressing materials such as fabrics and many objects can act as RH buffers. These factors produce an RH band that can be obtained in a particular showcase. When using dried silica gel (the most common control method for dry showcases required for archaeological iron or copper alloys), there is another consideration. The lifetime of the buffer, before it requires changing for dried buffer, depends on the showcase parameters, the initial RH (how well the silica gel was dried) and the final RH. Adding silica gel with a mid RH, e.g., 30% to avoid damage to organic artefacts and having to keep the RH below 35% for archaeological copper alloys, means the silica gel will need to be replaced very frequently.

The low RH requirements of archaeological iron and copper alloys mean they generate the most difficulty in display with organic materials that require higher RH values. Further work on iron was undertaken to confirm akageneite as the main damaging species. As the threshold RH values are critical, the 30% value selected within English Heritage was further explored. Archaeological materials are often significantly transformed during burial. Glass and bone were selected for study due to the amount of damage observed to these materials in English Heritage’s collection. English Heritage runs a decennial condition audit, inspecting 5% of objects on display and 1% in storage. Recent damage (in the last 10 years) is assessed. Glass and bone were the most frequently damaged archaeological materials after iron and copper alloys in the 2021 audit. Historical wood and leather showed more instances of damage, but not from the archaeological collections.

Showcases can be split physically and controlled passively as two separate compartments. This has been used successfully several times within English Heritage displays, with different designs. The glass separation can be visually intrusive. Mechanical control can be designed to provide different RH environments in different volumes of a single showcase, but this is relatively expensive and has long term maintenance and sustainability issues. The room environment and showcase properties will determine the RH band a particular showcase can maintain in that space.

Where a single RH or more realistically a RH range is used, the band can be determined from the RH reaction curves for both materials. The minimum damage can be determined from the overlap of the two curves, bearing in mind the difficulty of comparing damage to two different materials. There are positive data for the use of historical wood and parchment (HERIe). However, archaeological materials are often significantly changed from their original state and react differently. There is limited research on their response, with most guidelines resembling 45–55% for organics [22,23]. For archaeological bones, a single isotherm has been published in the grey literature [24], and Turner-Walker has shown that the strength of archaeological bone is related to the time buried [25]. A full understanding of degradation is complex and requires further understanding of water vapour isotherms, moisture content to expansion coefficients, modelling the water vapour ingress and take up, and mechanical properties evaluation (most authors set the limit at the limit of elastic deformation). Measuring the isotherm is a direct way to understand the response to moisture vapour. Archaeological bone is also a very heterogeneous material, and these properties are likely to vary across and through an object, increasing the research required. Whilst such extensive work is beyond the scope of this paper, the measurement and examination of moisture sorption isotherms can provide some useful initial information for risk assessment. Research has shown the deterioration rate of archaeological glass increases with increasing RH [26,27]. This is due to the process of hydration that gradually transforms it into an altered or ‘gel’ layer. At the same time, the pre-existing altered layer on corroded glass is vulnerable to dry conditions which cause its dehydration and subsequent cracking terminating in delamination through a phenomenon known as ‘crizzling’. The exact RH balance between these two extremes has been the topic of decades of research and is unknown thus far. This is partly due to the difficulty of empirical detection of the onset of crizzling. The consensus settles at approximately 35–50% RH for most glass and closer to 40% for more sensitive specimens [28]. However, these figures are often based on observational studies or, in older literature, the deliquescence RH (DRH) of single salts like sodium carbonate that can be found on corroded glass surfaces [29]. For mixtures with archaeological iron and copper alloys, the main question becomes what is the lowest RH that a glass can be exposed to and how damage increases with reducing RH. Again, the relevant research is not published, but examining moisture sorption isotherms can provide useful insights.

The DVS measurement can also investigate the kinetics of the sorption or desorption. The time at each RH can be fixed or determined by the rate of change in mass against time. A threshold value can be used to move to the next RH point. The examination of the data can produce a hygrometric half-life or response to 63.2%. Such data can be used to assess the impact of an environment on objects. Fluctuations in RH that are too fast will not allow the object to fully respond and can have little impact. Two examples of the response rate of objects were assessed, glass crizzling via visual observation under microscopy and ivory response via acoustic emission.

## 2. Materials and Methods

### 2.1. Archaeological Iron

Risks to archaeological iron come from a number of different reactions. At low RH values, the creation of akageneite and the expansion this creates is probably the highest risk mechanism. Over 1500 instances of deterioration from the collections of English Heritage and the British Museum were examined to determine the cause. Material was removed from the deterioration centres and analysed with FTIR (Nicolet Avatar 360, Waltham, MA, USA and Bruker Alpha, Billerica, MA, USA), Raman (Horiba Jobin Infinity, Kyoto, Japan) and X-ray diffraction (Phillips 1830/1840, London, UK and Bruker D8 advance, Billerica, MA, USA, 40 V, 40 mA). Mixtures of iron and iron (II) chloride powders in polyethylene caps were exposed to a series of RH values generated above glycerol solutions in 300 mL Bernardin Mason jars [30]. A number of different measurements were undertaken on the powders and the jar environment.

The mass gain was measured periodically with an EKG balance. Measurements were undertaken in the polyethylene cap.

The amount of akaganeite formed was determined with FTIR spectroscopy in potassium bromide discs. A sample of 3 mg of powder was removed and added to 300 mg potassium bromide and double pressed into a disc. This was analysed with a Nicolet Avatar 360 spectrometer using a published calibration with the 852 cm^−1^ absorption peak [31]. 

The water content was also extracted from the absorption band at 1650 cm^−1^. Calibration was undertaken by adding water from a micropipette to iron (II) chloride powder before producing KBr discs. 

The water content of the powder mixtures (separate sample) was also analysed with thermogravimetric analysis (Perkin Elmer TGA7 with an open platinum crucible, Waltham, MA, USA). The sample size was approximately 3 mg, and all samples were ground in a pestle and mortar. The furnace was flushed with 60 mL/min of zero grade nitrogen). 

The oxygen amount in the container was measured with a Presens 4 Oxygen metre with Presens Sp-PSt3-NAU-D7-YOP self-adhesive oxygen spots (Regensburg, Germany). 

The pressure generated by the reaction was measured in a sperate experiment by placing the powder mixture in a FTIR KBR disc press. The press was placed in a polycarbonate chamber with a glycerol solution to control the RH accurately [30]. The steel plunger of the press was placed against a linear variable displacement transducer (GTX1000, Stamford, CT, USA). The LVDT output was recorded with a SR008 voltage logger (Wilmington, MA, USA), recording any expansion. The experimental setup is shown in Figure 1.

### 2.2. Archaeological Bone

At English Heritage archaeological unworked animal bones are stored together in self-seal polyethylene bags in cardboard boxes. The boxes contain large numbers of bones from the same site and sometimes mixed context. The bags frequently have small fragments present. A selection of these were selected for DVS analysis. Fragments were selected from a series of sites identified as representative during PhD research [32]. Dynamic Vapour Sorption was carried out on a Surface Systems Adventure 1000 system (Buckinghamshire, UK).

Cracking was observed by eye on an archaeological bone plaque in a showcase at Chester Roman Fort. The plaque was Roman and, in the Bone, Shale, and Jet case, in the main room. The investigation of the environmental monitoring (Rotronic Hygroclip sensor, Bassersdorf, Switzerland) in the showcase indicated RH reductions of 63 to 33%. The cracking process was investigated with acoustic emission (Physical Acoustics Pocket AE with WD sensors). The plaque was placed on the sensor on a bed of cyclododecane. This material has been found to be a reasonable couplant and will fully sublimate, leaving no residues. The plaque’s RH response rate was investigated in a polycarbonate chamber controlled with glycerol solutions in a series of static air experiments, with RH drops between 63 and, 50, then 48, 46, 44, 42, 40, 38, 36, and finally 34%. The polycarbonate hood sat on a pressed 2 mm steel chamber with separate door and perforated steel plate to the polycarbonate chamber. The RH drop was induced by changing the glycerol solution through the separate door. The mass and acoustic emission were continuously monitored. The testing was stopped at 34% when acoustic emission increased. Mass was recorded on an EKG balance read via parallel port. Temperature was not controlled, beyond the winter heating at Corbridge store. The glycerol solution RH is not temperature dependant between 5 and 60 °C [25].

### 2.3. Archaeological Glass

To achieve a better understanding on the reaction of altered glass to changing RH, an array or glass samples was tested using the DVS Adventure vapour sorption analyser. All samples used in these experiments were fragments of authentic archaeological glasses from a representative sample of time periods. The glasses vary in their composition, morphology, and extent of corrosion. Their responses to changing RH would, therefore, also be expected to have notable differences in the same way that such glasses are broadly classed into distinct sensitivity levels for display and storage conditions. 

The samples from Apollonia (Arsuf, Israel; 656, 2009) are Byzantine 6–7th century CE colourless bowl rim fragments. The average composition of the excavated assemblage is shown in Table 1 and is typical for the soda–lime–silica glasses of this period. They are lower in soda than the those of the Roman period and, therefore, slightly more chemically durable. Such glasses are typically considered less sensitive to their environment and survive in relatively good condition considering their age. A full overview of the glass found at this site can be found in paper by Freestone et al. [32]. The analysis of sample No.67 included both the bulk glass and its brittle corrosion layer, while only the delaminated corrosion layer was analysed for glass No. 61.

The Medieval glass sample BV4 (794,981) from Battle Abbey is a fragment of a corroded potash–lime–silica glass vessel with a > 150 µm altered layer with surface mineral deposits. During excavations, glass from this or several preceding centuries is typically found in very poor condition and, in many cases, consists entirely of an altered layer with a few millimetres of original glass inside. It requires specialist treatment and well-controlled storage due to its friability and high sensitivity to RH fluctuations. This 15–16th century shard was never truly underground and was instead buried under thick rubble from the time of the Dissolution of the Monasteries, hence explaining its relatively good condition. A more detailed description of the glass found at Battle can be found in a report by compiled by Hare [33] (pp. 127–147). Vessel glass from Battle was treated by immersion in 4-3-2 glass mix (with Vinamul 6815 emulsion polymer), rinsed and left to dry, then painted with c.25% PVA. It is unknown whether this treatment was also applied to the studied sample. 

The MDBA 615 7B Topsoil window glass and the White window glass originate from the 2002–2005 Beeleigh Abbey excavation. They lacked a definite context as they were found in a single large surface deposit, likely originating from the destruction caused by the Dissolution of the Monasteries. The latter samples were an assortment of small (<1 cm), severely corroded, and unidentifiable fragments. The former was a sample of a c. 3–4 cm wide window glass shard with grisaille decoration and its bulk composition analysis results are available in Table 1. It is a specimen of high-lime, low-alkali (HLLA) glass, most likely from the first half of the 16th century. Such glass is chemically more durable than its Medieval potash–lime–silica predecessors. As most other analysed glasses from this assemblage were of the same type, it can be assumed that the unsorted Topsoil glass is also HLLA fragments. The glass was previously stored in uncontrolled conditions in an exposed environment.

The Brown and Pink early Post-Medieval glass samples are unidentified archaeological glass fragments from Northern Europe. Their composition is unknown but likely to be similar to Beeleigh Abbey glass. They were mostly stored in uncontrolled indoor environments and exhibit a medium level of corrosion (c. 30–80 µm, flaky altered layer). 

All samples were small individual glass chips of <150 mg except for the Beeleigh Abbey topsoil fragments that collectively totalled c. 650 mg. All glass samples were subjected to 0–95% RH cycles to obtain a complete isotherm. The potash–lime–silica glass BV4 (794,981) was tested two more times using four cycles of 20–60% RH at 20 °C and 30 °C to examine the changes in its response to the same conditions. The surface area of the samples or the proportion between the altered layer and the base glass were not measured for these experiments, and therefore, the results are not directly compared between glass types based on the percentage of the mass gained. Instead, the conclusions are drawn from the shape of each isotherm. 

A separate study was conducted on pieces from a plate of archaeological window glass that were observed to have white crystals on their surface during conservation. Analysis was requested to identify these. The pieces were transported in a polypropylene box with silica gel conditioned to 45% RH. The pieces were examined under a microscope (with fibre optic lighting to avoid heating, which has been observed to cause damage to some glasses). New small-scale cracks were observed within 3 min of removal from the boxes. The room RH was found to be 32%, measured with Hanwell radio telemetry system. The examination of the storeroom RH indicated it had not dropped below 45% in the past 45 years, measured with Hanwell radio telemetry system and thermohygrographs. Both types of monitoring had RH calibration checked at three points annually. The response of the pieces when the RH dropped from 45 to 32% was investigated by weighing pieces (EKG balance) read via parallel port in a Binder environmental chamber at 25 °C. The measurement was also repeated in static air, using a similar method with the balance and glass sample in a polycarbonate chamber, conditioned with glycerol solutions. The glycerol solution was continuously stirrer with a magnetic stirrer. The polycarbonate hood sat on a pressed 2 mm steel chamber with separate door and perforated steel plate to the polycarbonate chamber. The chamber was placed in the environmental chamber to control temperature. Several pieces were available and of limited archaeological value. A cross section was produced and examined in SEM-EDX. The alteration layer was found to be over 80 micrometres thick. Vickers hardness fracture toughness measurements were undertaken on the cross sections with an indentor at 19.6 N force, producing approximately 40-micrometre indentations. With the limited alteration depth present, the cracks induced from the two diamond corners parallel to the surface only were used. The cracks generated perpendicular to the surface sometimes reached the outer surface or inner glass surface. The length of the radial cracks parallel to the layer was measured, and the fracture toughness was calculated [34].

## 3. Results

### 3.1. Archaeological Iron

**Figure 2:**
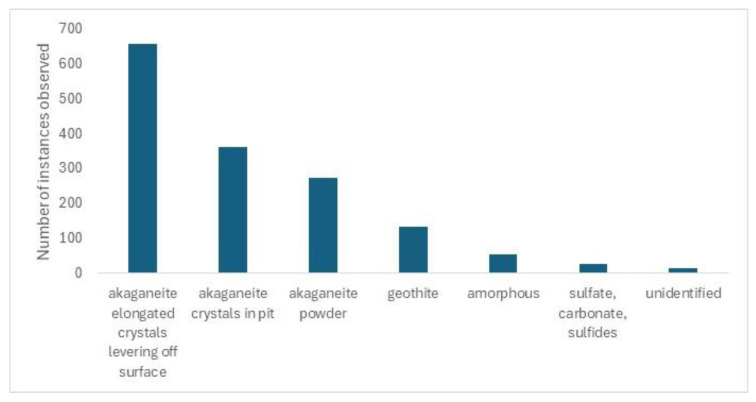
Species analysed at corrosion centres on deteriorating archaeological iron.

The analytical results from the over one thousand examples of deteriorating archaeological iron are shown in Figure 2.

Akaganeite appears to be instrumental in 84% (1292/1532) of the instances of deterioration observed.

**Figure 3:**
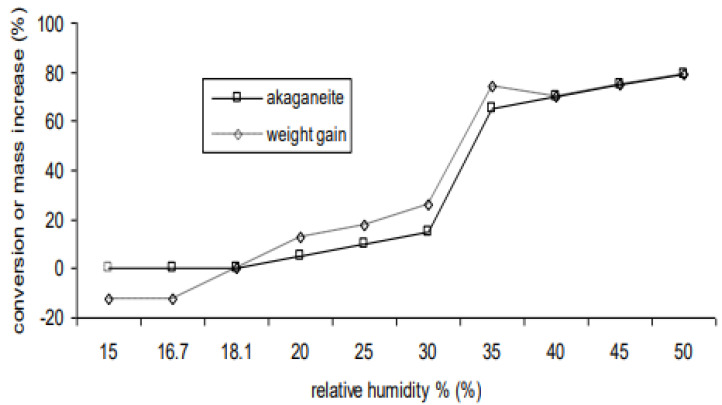
Amount of mass gain and akaganeite formed from iron and ferrous chloride powders after 24 months at various relative humidities.

The amount of akageneite formed increases by four times between 30 and 40% RH. Comparison of the recorded mass gains with the FTIR data after 24 months exposure shows some discrepancy in Figure 3. Both the amount of akaganeite and the mass gain were scaled as a percentage of the original mass of iron present in the sample for comparison. 

There are quite large discrepancies between the two traces. Below 18% RH, the powder lost approximately 12% mass. This is due to the previously recognised conversion of FeCl_2_·4H_2_O to Fe_2_Cl_2_·2H_2_O [16]. Between 20 and 40%, the mass gain is somewhat higher than the amount of akaganeite detected by FTIR. Table 2 shows the water content as measured by FTIR and TGA. 

This correlates well to the differences between the measured mass gain and the mass gain expected from the amount of akaganeite formed. These values are the combined mass of water due to surface adsorption and waters of hydration in the ferrous chloride. At 15% RH, the powder mixture contained 10% water by mass due to the two waters of hydration of Fe_2_Cl_2_·2H_2_O. At 20% RH, Fe_2_Cl_2_·2H_2_O gains two extra waters of hydration to form Fe_2_Cl_2_·4H_2_O, and a small amount of akaganeite has formed. The mass of the sample has increased by 13%, 8% greater than is accounted for by the mass increase due to akaganeite. The discrepancy increases to a maximum at 30% RH and decreases to almost zero by 40% RH. Almost all of the water content is due to adsorbed water at this RH and above. The adsorbed water masks the increase in akaganeite formation between 30 and 35% RH in the mass data (Figure 1). This difference between the amount of akageneite formed and mass gain is important as other authors have assessed risk based on mass gain measurements [5,14,15].

**Figure 4:**
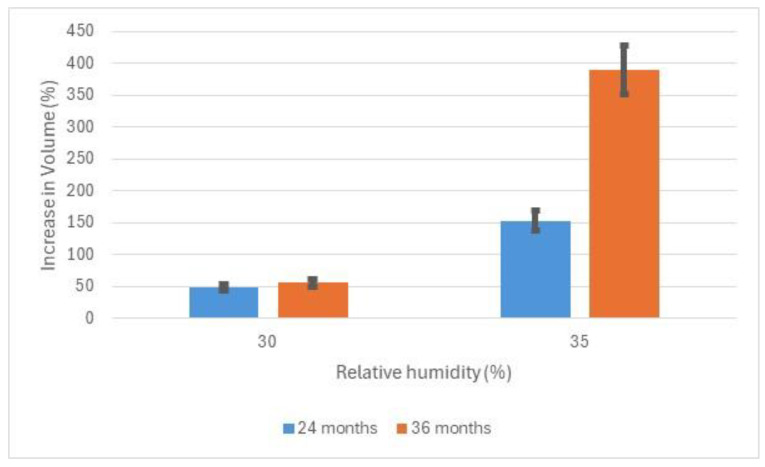
Increase in volume of iron and ferrous chloride powders after 24 months and 3 months at low relative humidities.

Figure 4 shows the volume increase in iron and iron (II) chloride powder mixtures at different RH values.

**Figure 5:**
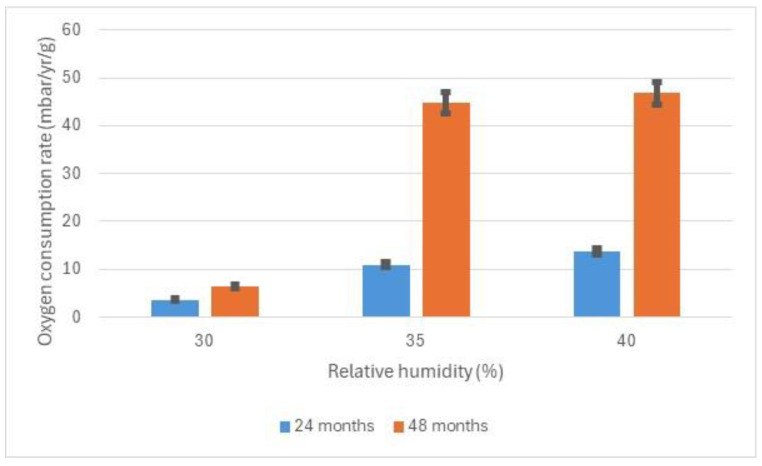
Results of oxygen consumption studies for iron and ferrous chloride powders after 24 months and 36 months at low relative humidities.

As akageneite is formed, the elongated crystals exert pressure on the transducer and the volume of the sample expands. The volume increase is calculated from the height increase in the powder mixture. There is a dramatic increase in expansion between 30 and 35% RH after 24 months. This increases much further after 36 months. The volume expansion correlates well with observation of deteriorating archaeological iron. Objects were frequently observed with the surface being levered off with elongated crystals or even severe delamination with elongated akageneite crystals pushing the pieces apart. Figure 5 shows the oxygen consumed during exposure of the powders to various RH values for 24 months and 48 months.

After 24 months, over four times as much oxygen has been consumed at 40% compared to 30% RH. After 48 months, this ratio has increased to almost nine.

The measurements indicate a variety of increases in rate between 30 and 35 or 40% RH, depending on the parameter measured. They are consistent with the concept that the risk is related to the amount of akageneite formed. Measuring mass can be misleading in this RH region due to extensive water uptake occurring simultaneously with akageneite formation. The differences between 30% and 35 or 40% increase with increasing exposure time. These longer time rates are probably more relevant to the object lifetime.

### 3.2. Archaeological Bone

**Figure 6:**
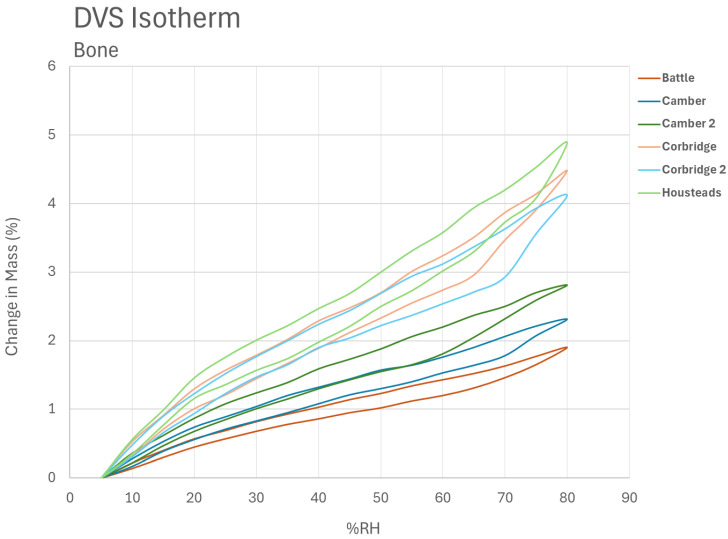
The DVS isotherms for the bone fragments tested in this study, coloured by site. The desorption curve always remains above the absorption curve.

The isotherms of the eight bone fragments are shown in Figure 6. 

The maximum amount of absorbed water decreases across the samples. Comparison with the analysis on bones from those sites by FTIR and neutron and X-ray diffraction shows this follows the deterioration ranking [32]. Bones from Housteads Roman Fort were assessed as the least degraded, followed by Corbridge Roman City, then Camber Castle, with those from Battle Abbey being the most degraded.

The bones all show very wide hysteresis, when the sorption and desorption bands do not coincide over almost all of the RH range measured. The hysteresis bands are closer together in the more degraded bone and further apart in the bone fragments from Housteads and Corbridge. The isotherms steepen on desorption below 25% RH and on sorption above 75–85%.

The changes in response are probably related to the digenesis of the bones. The more the diagenesis occurs (dependent on time of burial and soil conditions), the more altered the bone is. The amount of intact collagen reduces, porosity develops, and pore size distribution changes. Soluble salt and other species are introduced during burial. Turner-Walker showed mechanical properties reduce as diagenesis progresses [25].

The bone fragments only form part of the thickness of the bones they originated from. Hence, the response times measured are almost certainly lower than would be expected from the whole bones. Archaeological bone is a very heterogeneous material [32,35,36], often with significant differences within a single bone. A large number of analyses will be required to represent the full set of bone material present in a collection. Measurements will continue to provide a better distribution of the responses of bones in the collections English Heritage cares for.

**Figure 7:**
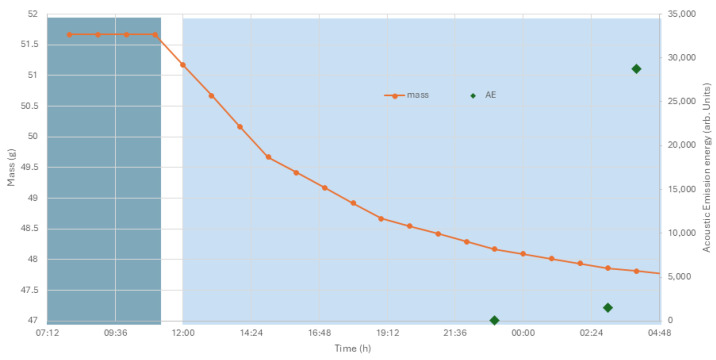
Mass and acoustic emission energy from the bone plaque when RH drops from 63% (darker blue box) to 32% (paler blue box). The RH drop took place over a two-hour period.

The response of the archaeological bone plaque and acoustic emission observed is shown in Figure 7.

The mass response is relatively slow, taking several hours. Acoustic emission, indicating micro-fracturing, begins only towards the later part of the response. 

### 3.3. Archaeological Glass

The isotherms of the archaeological glass samples are shown in Figure 6 displaying both the full RH scale (0–100%) and a truncated scale for better representation of the RH levels typically found in indoor environments (20–60%). In general, all samples exhibited a notable and asymmetrical response to the changing RH as well as considerable hysteresis between the absorption and desorption cycles. The magnitude of this difference was largely proportional to the thickness of the glass altered layer and surface mineral deposits. Across the entire sample assemblage, there was also a trend in the differences between the absorption and desorption isotherm curve shape. The response time was relatively slow, with equilibration typically achieved after 30 min to several hours of exposure to a change in RH. It is also worth noting that the equilibration times were also only slightly longer for larger as opposed to small RH changes.

**Figure 8:**
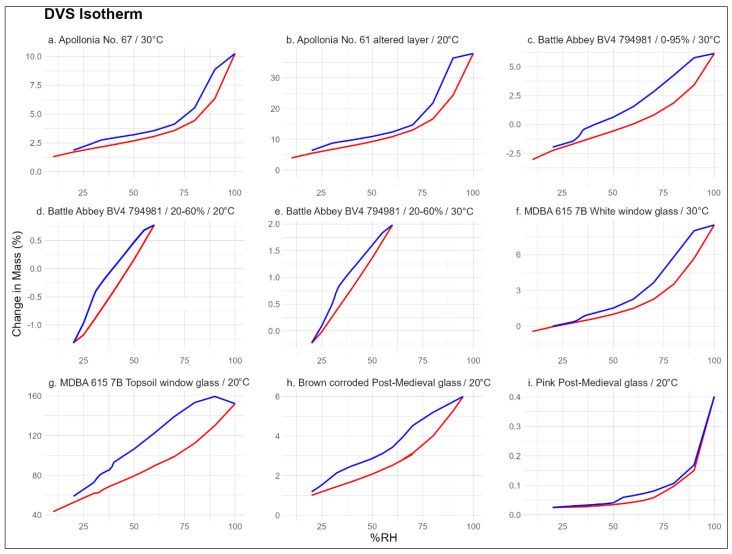

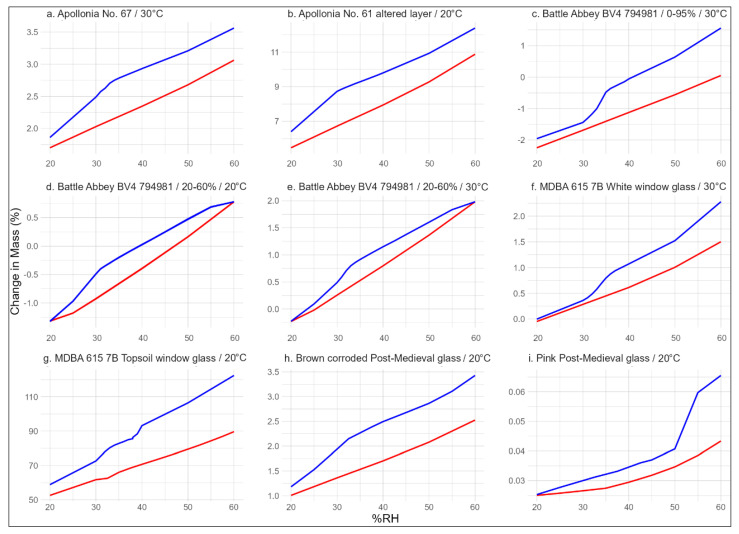
The isotherms of the studied glass samples at 0–100% (**above**) and 20–60% (**below**) RH scales. The red line represents sorption and blue line represents desorption. The sample name and temperature used for the experiment are indicated above each plot (**a**–**i**). Test (**a**) was performed on a Byzantine soda–lime–silica glass sample and (**b**) on the altered layer of another glass of the same type. Tests (**c**–**e**) were performed a Late Medieval potash–lime–silica window glass chip. Tests (**f**–**i**) were conducted on Northern European Post-Medieval, likely soda–lime–silica glass.

In terms of absorption, the sample mass increases at a steady rate between approximately 10 and 60% RH, then exhibits an upturn as the RH increases further, should in Figure 8. The extent of this rate increase varies between samples, but in some cases, the 60–95% range can account for over half the mass gain of the cycle. On the other hand, the desorption can be split into three stages. Initially, the mass drops sharply between c. 95 and 60% RH, albeit never reaching the levels seen during absorption. The mass then continues to decrease steadily until it reaches another threshold at 25–35% RH. The isotherms exhibit a small but relatively sharp drop that is observable in all samples but can be seen particularly well in specimens from Apollonia and Battle Abbey. Once it begins, this drop usually occurs over a further RH decrease of 4–6%. Following this, the mass settles and continues to decrease, eventually equalising with the sorption mass at 10–20% RH. The exact positioning of this drop depends on the sample and on the temperature of the experiment. For instance, during the 20–60% RH cycling study for BV4 (794,981) glass, the drop commenced at 31–30% RH at 20 °C, while during an otherwise identical experiment at 30 °C, it commenced at 33–32% RH.

These patterns occur over all the studied glass samples to various degrees and could have significant implications for corroded historical and archaeological glass storage. The large mass fluctuations and hysteresis occurring after 60–65% RH signal that such humidity levels should ideally be avoided for all types of glass. In fact, the amplitude of mass fluctuations of BV4 (794,981) glass subjected to repeated 20–60% cycling only reached c. 2.5% compared to the c. 8% difference between 20% and 95% RH. For chemically unstable glass, such fluctuations could mean cycling between alteration layer growth and its physical breakdown, resulting in a gradual loss of surface features or even a complete collapse of the object. For more hydration-resistant glasses such as the Byzantine specimens from Apollonia, the growth of the alteration layer in static humid conditions is of lesser concern. However, fluctuations could cause its existing corroded surface layer to delaminate due to stress. This is reflected in the results of the experiment conducted on the detached altered layer sample of vessel No. 61, which experienced mass changes of nearly 40%. Its isotherm shape was also similar to that of the fragment of vessel No. 67 with its altered layer still attached, suggesting that the corrosion layer buffers a large proportion of the RH fluctuations. The 25–35% RH threshold is not as significant in terms of mass loss during dehydration. However, crossing it could also pose a danger to more sensitive glasses as this transition may denote a physical transformation process of with effects of an unknown magnitude. 

Despite their intriguing replicability, the avenues for interpretation of these findings are currently limited. DVS only collects mass change data but provides no indication on whether the water vapour was absorbed into the material or simply adsorbed onto surface pores with no effect on the sample. It is also unclear to which degree the vapour reacted with the altered layer or pristine glass as opposed to the superficial secondary mineral layer. However, the abrupt change in mass around 25–35% RH during desorption is unlikely to be associated with salt crystallisation as the same type of response is not observed on the absorption curve. A set of similar experiments was performed by Bellendorf et al. [36] by decreasing the RH of an environmentally controlled room in stages of 85%, 70%, 50%, and 30% while measuring the mass change with balances. It was found that the extent of the mass loss was proportional to the thickness of the altered layer, but the exact response to each RH stage depended on its morphology and the composition of the secondary mineral layer. Similarly to the results seen here, the greatest mass loss occurred between 85 and 50% RH. However, an artificially corroded model glass with a thick syngenite and gypsum crust experienced most of its mass loss between 85 and 70% RH, while the mass loss of a Medieval archaeological specimen was more evenly distributed between the 85 and 70% and 70 and 50% stages. This suggests that hydrated salts deposited on the altered are likely to play a part in the glass response to its environment.

**Figure 9:**
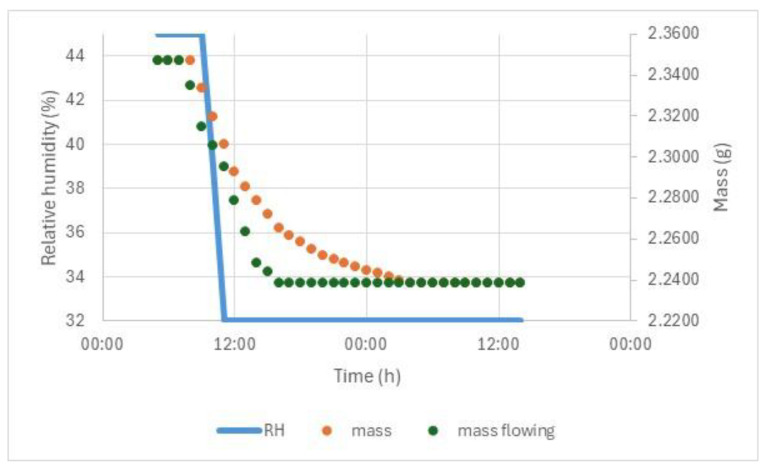
Mass of glass piece when exposed to RH drop from 45 to 32% (blue line) in still air (orange datapoints) and flowing air (green datapoints).

In turn, the response of the archaeological glass piece plaque is shown in Figure 9. 

The response is relatively slow, taking several hours. This is unlike the very rapid observed cracking. The fracture toughness was measured to be 0.04–0.08 MPa√m. This is extremely low, as most glasses measure between 0.54 and 0.87 MPa√m [37,38]. The indentation crack technique used has several limitations [39], and the results were variable across the alteration layer. However, the order of magnitude of the result is probably reasonable.

## 4. Conclusions

The additional measurements at 30, 35, and 40% RH have indicated the change in risk to archaeological iron increases dramatically from 30 to 35% RH. To some extent, reinforcing 30% as a level below is sufficient to keep and maintain such a material. This re-emphasizes the need for knowledge of the lower RH tolerance of other materials to be displayed alongside iron in a showcase. The inspection of the measured isotherms for archaeological bone and glass has generated some useful insights into their likely behaviour. For bone, the isotherms indicate that efforts to avoid dropping below 25% RH will be beneficial from observations on a plaque and in previously reported research on objects [39]. The isotherms of the archaeological glass measured indicate avoiding RHs above 60% and below 35 to 30%. However, observations indicate rapid cracking at timescales well under the equilibrium time needed for the whole alteration layer. This may be related to the very low fracture toughness of the alteration layer, with tiny cracks initiating at the surface rapidly and extensive cracking then progressing. Some care should be exercised in using these isotherm results, as the mechanical behaviour is much more complex. But, in the absence of a full analysis, their use can be beneficial for preliminary risk assessment for mixed showcases. The information gained has been used to design several mixed archaeological bone and iron or copper alloy showcases. These have been successfully controlled for several years to between 25 and 30% RH with dehumidifiers or 25 and 35% with silica gel and low air exchange rate showcases. A mixed approach with a dehumidifier in one showcase, controlled to 25–30% for iron and bone providing silica gel, to be swapped into a second showcase, with archaeological copper alloys and bone (30–35%), has also been successfully used. Further analyses with a higher number of glass samples from English Heritage collections and supplementary techniques are planned to further explore these phenomena, their causes, and implications for conservation practice.

## Figures and Tables

**Table 1:** The known chemical compositions of the glass samples in the DVS study collected using SEM-EDX measurements on pristine glass.

Oxide wt%	Na_2_O	MgO	Al_2_O_3_	SiO_2_	P_2_O_5_	SO_2_	Cl	K_2_O	CaO	TiO_2_	MnO	FeO	BaO
Apollonia 61 and 67 (avg.)	14.5	0.7	3.0	70.8	0.1	0.2	0.8	1.0	8.2	0.1	n.d.	0.5	n.d.
Battle BV4 (794,981)	2.57	6.38	1.80	58.41	3.30	n.d	0.44	10.01	15.55	n.d	0.83	0.72	n.d.
MDBA 615 7B White No. 1	2.68	3.21	1.49	58.98	2.98	0.62	0.47	3.51	24.55	0.17	0.71	0.51	0.12

**Table 2:** Water content as measured by FTIR and TGA.

Relative Humidity	Water Content (% weight/weight of Sample)		Difference Between Mass Gain Measured and That Predicted from the Amount of Akaganeite (% weight/weight of Sample)
	TGA	FTIR	
15%	10.0	10.0	0 *
20%	22.1	22.3	8
25%	19.3	19.5	8
30%	14.3	14.5	11.2
35%	9.5	9.3	9
40%	1.1	0.8	1.0
50%	0.3	0.4	0.2
70%	0.2	0.1	0.1

* at 15% RH no akaganeite was detected, there was a mass loss of 12% due to dehydration of FeCl_2_·4H_2_O to FeCl_2_·2H_2_O.

## Data Availability

The raw data supporting the conclusions of this article will be made available by the authors on request.
